# Pancreatitis Associated With Teduglutide: A Disproportionality Analysis via the Food and Drug Administration Adverse Event Reporting System (FAERS) Database

**DOI:** 10.7759/cureus.68091

**Published:** 2024-08-29

**Authors:** Joyce H Gu, Zachary Sheingold, Mark Samarneh

**Affiliations:** 1 Medicine, Lake Erie College of Osteopathic Medicine, Greensburg, USA; 2 Internal Medicine, St. John's Riverside Hospital, Yonkers, USA; 3 Internal Medicine/Nephrology, Riverside Health System, Yonkers, USA

**Keywords:** food and drug administration adverse event reporting system (faers), pharmacovigilance, disproportionality analysis, medication-induced pancreatitis, teduglutide

## Abstract

Introduction

Teduglutide is a glucagon-like peptide-2 analog that is indicated for the treatment of short bowel syndrome (SBS) by reducing patient dependence on parenteral support. Due to the rarity of SBS as well as the recent timeline of the adoption of teduglutide, the safety of teduglutide is relatively poorly understood. Several recent clinical case reports have highlighted elevated pancreatic enzymes and pancreatitis as a concerning complication of teduglutide. This prompts a systematic study of the association between pancreatitis and teduglutide.

Methods

This study conducts a case-control design disproportionality analysis by using data from the US Food and Drug Administration Adverse Event Reporting System (FAERS). Reports from the first quarter of 2020 through the first quarter of 2024 were retrieved from this database, and a disproportionality analysis was conducted. The analysis consisted of traditional methods of analyzing adverse drug events such as the reporting odds ratio (ROR) and proportional reporting ratio (PRR), as well as Bayesian methods such as the empirical Bayes geometric mean (EBGM) and information component (IC). A confidence interval for ROR and PRR that excludes a ratio of 1 or a confidence interval for IC that excludes a score of 0 was used as the criterion for a statistically significant association between pancreatitis risk and teduglutide use.

Results

Out of 11,696 reports of teduglutide adverse effects in over four years of adverse effects data drawn from the FAERS database, 79 cases of pancreatitis were identified. The disproportionality analysis revealed an ROR of 3.73 (95% CI (2.99, 4.66)), a PRR of 3.71 (95% CI (2.97, 4.63)), an EBGM of 3.70, and an IC of 1.84 (95% CI (1.51, 2.16)). All of these statistics indicate a statistically significant association between pancreatitis risk and teduglutide use.

Conclusion

The results reveal a statistically significant association between pancreatitis risk and teduglutide use. Our findings highlight the necessity for the careful monitoring of pancreatitis in patients undergoing teduglutide therapy going forward.

## Introduction

Short bowel syndrome (SBS) is a disease that occurs when the length of the functional small bowel is too short to sustain adequate nutritional absorption, commonly due to extensive surgical resection or congenital defects. Such surgical interventions are seen in the management of various severe small bowel disorders including Crohn's disease, enterocolitis [1], vascular disease, volvulus, cancer [2], and radiation enteritis [3].

Due to the severely decreased functional capacity of the short bowel, SBS patients become dependent on parenteral nutrition to meet the necessary nutritional requirements [1]. However, parenteral nutrition is a less-than-ideal management plan for this condition, due to various risk accompanying risk factors such as catheter occlusion and infection, mechanical issues, central vein thrombosis, and metabolic complications [4].

Teduglutide is a drug that has been recently introduced to reduce parenteral nutrition requirements in SBS patients. Approved in the United States by the Food and Drug Administration (FDA) for clinical use in 2012, teduglutide is a recombinant analog of the natural hormone glucagon-like peptide-2 (GLP-2), which is secreted from intestinal cells to stimulate intestinal blood flow, mucosal proliferation, and nutrient absorption. The activation of GLP-2 receptors in enteroendocrine cells, myofibroblasts, and enteric neurons leads to the growth of the intestinal mucosa and the local release of insulin-like growth factor-1, nitric oxide, and keratinocyte growth factor [5]. Several studies have shown the benefits of teduglutide in reducing the volume of parenteral support required and in some cases even achieving full enteral autonomy [2,6-9].

While the aforementioned studies on the clinical benefits of teduglutide have generally reported that teduglutide is relatively well-tolerated, other literature on teduglutide have observed elevated amylase and lipase [10-13] and pancreatitis [6,11,14,15] as adverse outcomes of teduglutide. Jeppesen et al. conducted a randomized trial of 83 patients, in which one patient using teduglutide developed pancreatitis [6]. Conejo et al. gave a case report of a patient who developed pancreatitis following teduglutide administration, which resolved upon drug cessation [15]. Kochar et al. conducted a retrospective cohort study of 13 Crohn's disease patients on teduglutide therapy, of which one patient experienced elevated lipase and amylase and one patient developed pancreatitis [11]. Lee et al. gave a case report of a Crohn's disease patient using teduglutide who developed elevated lipase and amylase [12]. Kinberg et al. documented the case of a pediatric patient who discontinued teduglutide treatment upon developing pancreatitis [14]. Finally, Kim et al. reported that eight out of 11 patients receiving teduglutide experienced elevated lipase and amylase levels [13].

Despite the multiple reports of elevated pancreatic enzymes and pancreatitis associated with teduglutide usage, each of these reports consists of only a small number of patients, and there has not been a systematic large-scale study of the association between pancreatitis and teduglutide to date. Thus, we conduct a retrospective evaluation of this possible drug and adverse event relationship using the US Food and Drug Administration Adverse Event Report System (FAERS) database.

## Materials and methods

Data source

This study uses the FAERS database as the source of data [16]. The FAERS database is a publicly available database with quarterly data on spontaneous reports of adverse drug events ranging from 2012 to 2024. Data from the first quarter of 2020 (2020 Q1) until the latest available data from the first quarter of 2024 (2024 Q1) is included in the study. This data is downloaded in ASCII format from the FDA website and imported and analyzed using the Python programming language.

The FAERS database is composed of various data files that capture different aspects of adverse drug event reports. We primarily use the reaction (REAC) and drug (DRUG) files, which contain information about the adverse reaction and the drug. These data are associated together by the primary ID (primaryid) and case ID (caseid) fields, which uniquely identify the adverse reaction report. Cases pertaining to teduglutide were identified by querying the drug name (drugname) field for the generic name TEDUGLUTIDE and the brand name GATTEX, while cases pertaining to pancreatitis were identified by querying the preferred term (pt) field for Pancreatitis. While there could be multiple different drug reactions associated with each case, duplicate entries were dropped by using the unique identifiers primaryid and caseid.

Statistical analysis

The statistical analysis conducted in this study is based on data mining techniques for disproportionality analysis in pharmacovigilance [17]. These techniques give quantitative measures for detecting associations between a particular drug and a particular adverse drug event. All methods utilized in this work study statistics based on a contingency table that displays the prevalence of the adverse drug event of interest in the presence and absence of the particular drug and the prevalence of all other drug events in the presence and absence of the particular drug. The entries of the contingency table are commonly denoted as a, b, c, and d, as shown in Table 1.

**Table 1:** Contingency table used in disproportionality analyses, where entries a, b, c, and d are used in formulas for computing disproportionality statistics The letter a represents the number of adverse drug event reports that report pancreatitis associated with teduglutide use, the letter b represents the number of reports that report other adverse effects associated with teduglutide use, the letter c represents the number of reports that report pancreatitis associated with other drugs, and the letter d represents the number of other adverse events (besides pancreatitis) associated with other drugs (besides teduglutide).

	Pancreatitis	Other adverse effects
Teduglutide	a	b
Other drugs	c	d

The proportional reporting ratio (PRR) compares the percentage of pancreatitis events among teduglutide adverse events and the percentage of pancreatitis events among all other adverse drug events. The reporting odds ratio (ROR) captures a similar quantity expressed in terms of odds rather than percentages. These two quantities are often close when the number of observations is sufficiently large, which is observed in this study. The empirical Bayes geometric mean (EBGM) compares the percentage of pancreatitis events associated with teduglutide among all adverse events with this percentage assuming that pancreatitis risk is independent of teduglutide use. The information component (IC2) takes the base 2 logarithm of the EBGM. The confidence interval for IC2 takes a Bayesian approach which assumes a prior distribution on the occurrences of the adverse drug events, which makes it more appropriate when the number of observations is relatively small [17]. The formulas used to compute these statistics and their 95% confidence intervals are shown in Table 2.

**Table 2:** Formulas for disproportionality statistics and their 95% CIs The 95% CI for EBGM is not computed due to difficulty of implementation. The 95% CI for IC2 follows the formula given by Jiang et al. [18]. ROR: reporting odds ratio; PRR: proportional reporting ratio; EBGM: empirical Bayes geometric mean; IC2: information component; CI: confidence interval

	Statistic	95% CI
ROR	$\mathrm{ROR} = \frac{a/c}{b/d}$	$\exp\left(\log(\mathrm{ROR}) \pm 1.96\sqrt{\frac1a + \frac1b + \frac1c + \frac1d}\right )$
PRR	$\mathrm{PRR} = \frac{a/(a+b)}{c/(c+d)}$	$\exp\left(\log(\mathrm{PRR}) \pm 1.96\sqrt{\frac1a + \frac1b + \frac1c + \frac1d}\right )$
EBGM	$\mathrm{EBGM} = \frac{a(a+b+c+d)}{(a+b)(a+c)}$	
IC2	$\mathrm{IC}_2 = \log_2 \mathrm{EBGM}$	

## Results

Baseline characteristics

Some baseline characteristics of the demographic that reports adverse effects of teduglutide are included in Table 3. We note that not all patients represented in the database have reported their age and sex, so the numbers displayed in the table are restricted to the reports that do provide this information.

**Table 3:** Baseline characteristics of the demographic reporting adverse effects of teduglutide included in the study

Characteristic	Category	N (%)
Age (in years)	Less than 18	1,629 (20.4%)
	18-24	193 (2.4%)
	25-64	3,920 (49.2%)
	65 and older	2,229 (28%)
Sex	Female	6,723 (59.5%)
	Male	4,568 (40.5%)

We observe a bimodal distribution of ages in the population with peaks in pediatric patients and older adults. This is consistent with the fact that most cases of SBS are seen in either pediatric patients with congenital defects of the gut or older adults who suffer from inflammatory disorders such as Crohn's disease [1,2]. We also observe a higher percentage of females than males, which is consistent with the demographics observed in earlier randomized controlled studies of the effects of teduglutide [6-8].

**Figure 1:**
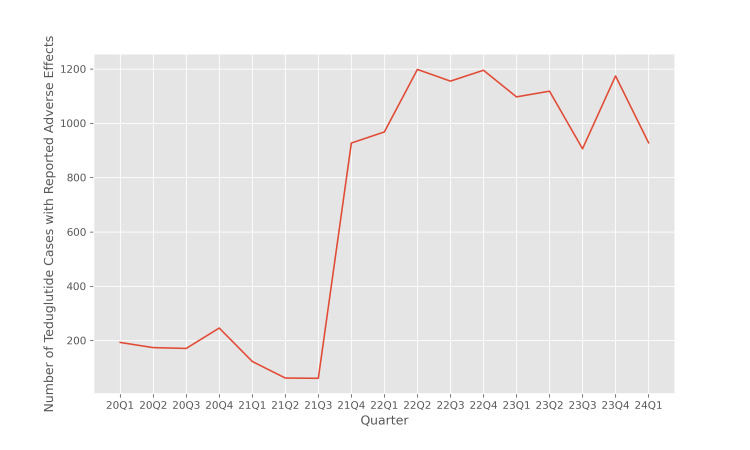
Number of teduglutide cases with reported adverse effects

Figure 1 illustrates the trend of the number of reports of adverse outcomes of teduglutide reported in the FAERS database in each quarter from 2020 Q1 to 2024 Q1. We observe a substantial increase in the number of reports of teduglutide in 2021, which may reflect a rise in popularity and widespread adoption of this drug for the treatment of SBS.

**Figure 2:**
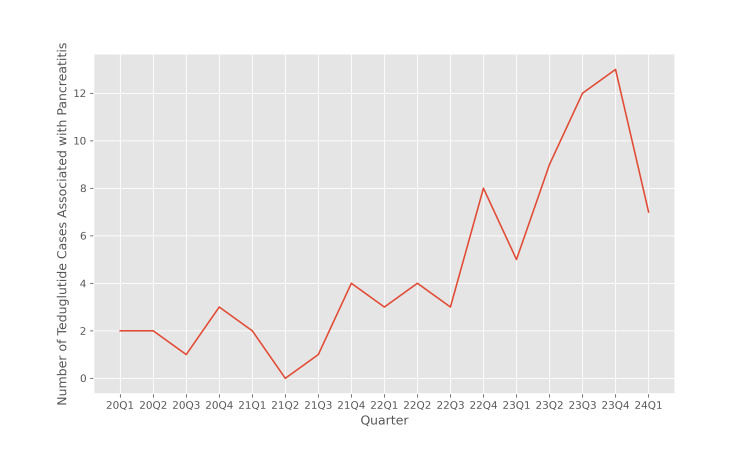
Number of teduglutide cases associated with pancreatitis

Figure 2 illustrates the number of reports of pancreatitis associated with teduglutide therapy in each quarter from 2020 Q1 to 2024 Q1. As in Figure 1, we observe an increasing trend in the number of pancreatitis event reports associated with teduglutide. To determine whether this increase in the number of pancreatitis reports is in proportion to the increase in the use of teduglutide therapy, we must conduct a disproportionality analysis. This investigation is carried out in the following sections.

Temporal trends of expected and actual cases of pancreatitis associated with teduglutide therapy

**Figure 3:**
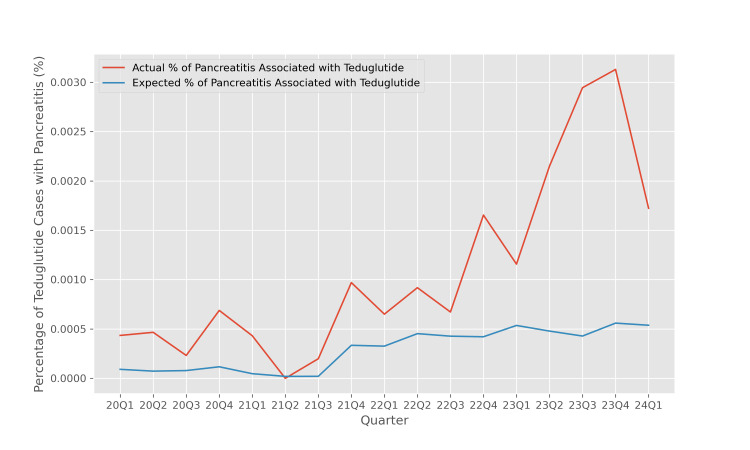
Actual versus expected percentage of teduglutide cases with pancreatitis The actual percentage of teduglutide cases associated with pancreatitis is computed as the number of teduglutide cases associated with pancreatitis divided by the total number of cases included in the study. The expected percentage of teduglutide cases associated with pancreatitis is computed by multiplying the percentage of all reports associated with teduglutide use and the percentage of all reports associated with pancreatitis.

One way of quantifying the disproportionality of pancreatitis cases is to compare the percentage of cases of pancreatitis associated with teduglutide therapy with the expected percentage of such cases assuming that pancreatitis risk is independent of teduglutide use. The ratio of these two quantities is the EBGM, and its logarithm base 2 is the IC2. Figure 3 illustrates the temporal trends of these two quantities across the quarters included in the analysis.

Disproportionality analysis of pancreatitis associated with teduglutide therapy

We conduct a disproportionality analysis of the association between pancreatitis and teduglutide by aggregating the adverse effects data drawn from the FAERS database from 2020 Q1 to 2024 Q1. The contingency table for this disproportionality analysis is shown in Table 4, and the results of the disproportionality analysis are shown in Table 5. We find that the association between teduglutide and pancreatitis has an ROR of 3.73 (95% CI (2.99, 4.66)), a PRR of 3.71 (95% CI (2.97, 4.63)), an EBGM of 3.70, and an IC2 of 1.84 (95% CI (1.51, 2.16)). All of these results indicate a strong association between teduglutide and pancreatitis. In particular, the PRR indicates that the patients undergoing teduglutide therapy are 3.71 times as likely to report a pancreatitis adverse event, compared to patients using all other drugs.

**Table 4:** Contingency table of teduglutide usage and pancreatitis

	Pancreatitis	Other adverse effects	Total
Teduglutide	79	11,617	11,696
Other drugs	13,673	7,499,604	7,522,917
Total	13,752	7,511,221	7,524,973

**Table 5:** Disproportionality analysis of pancreatitis associated with teduglutide usage ROR: reporting odds ratio; PRR: proportional reporting ratio; IC2: information component; CI: confidence interval

	Statistic	CI	Significance criteria
ROR	3.73	(2.99, 4.66)	CI >1
PRR	3.71	(2.97, 4.63)	CI >1
IC2	1.84	(1.51, 2.16)	CI >0

We additionally provide a stratified analysis by sex (Table 6) and age (Table 7). A combined ROR and PRR can be computed by aggregating across the stratification levels via the Mantel-Haenszel method for each of sex (ROR=3.69, PRR=3.67) and age (ROR=3.55, PRR=3.53), which gives similar results as our analysis without stratification. Thus, these results suggest that age and sex are not confounding factors in our findings.

**Table 6:** Contingency table of teduglutide usage and pancreatitis, stratified by sex

	Pancreatitis		Other adverse effects	
Teduglutide	Female	44	Female	6,716
	Male	30	Male	4,568
	Unspecified sex	5	Unspecified sex	404
Other drugs	Female	6,770	Female	3,780,862
	Male	5,066	Male	2,663,901
	Unspecified sex	1,876	Unspecified sex	1,071,339

**Table 7:** Contingency table of teduglutide usage and pancreatitis, stratified by age

	Pancreatitis		Other adverse effects	
Teduglutide	Less than 18	2	Less than 18	1,502
	18-24	1	18-24	195
	25-64	31	25-64	3,994
	65 and older	11	65 and older	2,278
	Unspecified age	34	Unspecified age	3,719
Other drugs	Less than 18	528	Less than 18	250,587
	18-24	295	18-24	153,402
	25-64	4,988	25-64	2,180,515
	65 and older	2,532	65 and older	1,605,348
	Unspecified age	5,369	Unspecified age	3,326,250

**Figure 4:**
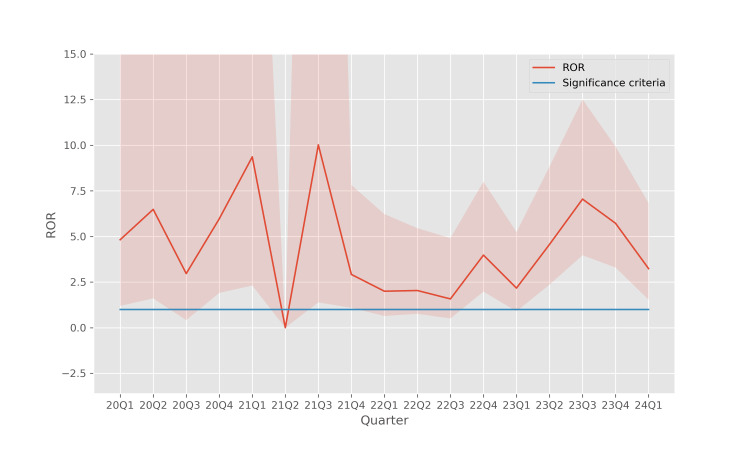
ROR of pancreatitis associated with teduglutide usage The shaded region indicates the 95% confidence interval for each quarter. ROR: reporting odds ratio

**Figure 5:**
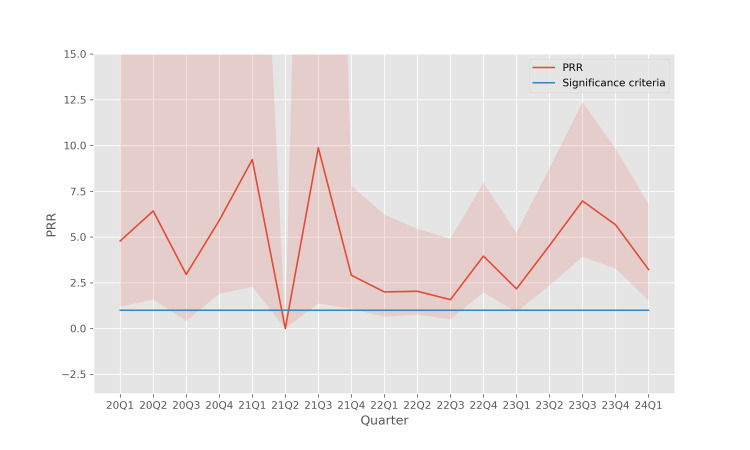
PRR of pancreatitis associated with teduglutide usage The shaded region indicates the 95% confidence interval for each quarter. PRR: proportional reporting ratio

**Figure 6:**
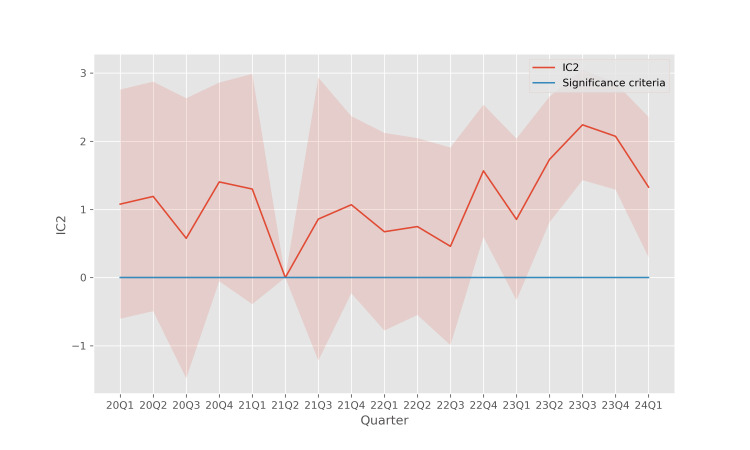
IC2 of pancreatitis associated with teduglutide usage The shaded region indicates the 95% confidence interval for each quarter. IC2: information component

The trends of the confidence intervals for these disproportionality metrics are shown in Figure 4 (ROR), Figure 5 (PRR), and Figure 6 (IC2). In these results, the disproportionality metrics are computed for each quarter, which reduces the sample size for the calculation and causes the confidence intervals to become wider. However, even with the reduced sample size, the analysis continues to reveal a significant disproportionality of occurrences of pancreatitis associated with teduglutide use, especially in the more recent quarters.

In earlier quarters, the sample size is relatively small which leads to wide confidence intervals which do not admit statistically significant conclusions. However, in more recent years, all three disproportionality metrics, ROR (Figure 4), PRR (Figure 5), and IC2 (Figure 6), support the fact that there is a disproportionate association between teduglutide and pancreatitis in individual quarters, with a stronger signal than is seen when these metrics are aggregated across all quarters.

Disproportionality analysis of other adverse effects of teduglutide

For comparison, a disproportionality analysis for other common adverse effects associated with teduglutide is conducted. The results of the study are shown in Table 8. Many of the other adverse events identified in our analysis are consistent with reports of adverse events in prior clinical trials [6-8]. In the top 10 adverse events of teduglutide by number of cases, the ROR ranges from 2.30 to 322.84, and the PRR ranges from 2.20 to 287.23. Pancreatitis has an ROR and PRR that is larger than four of these: nausea, diarrhea, vomiting, and product dose omission issue.

**Table 8:** Disproportionality analysis of the 10 most common adverse drug events associated with teduglutide ROR: reporting odds ratio; PRR: proportional reporting ratio; CI: confidence interval

	# of cases	ROR	95% CI	PRR	95% CI
Weight decreased	1439	8.45	(7.99, 8.93)	7.53	(7.12, 7.96)
Vascular device infection	1294	322.84	(301.52, 345.66)	287.23	(268.26, 307.53)
Product dose omission issue	1145	3.62	(3.41, 3.85)	3.37	(3.17, 3.58)
Diarrhea	1128	2.96	(2.79, 3.15)	2.77	(2.61, 2.95)
Abdominal pain	982	7.74	(7.25, 8.26)	7.17	(6.72, 7.66)
Nausea	945	2.30	(2.15, 2.46)	2.20	(2.05, 2.35)
Weight increased	920	6.67	(6.23, 7.14)	6.22	(5.82, 6.66)
Dehydration	842	14.00	(13.04, 15.02)	13.06	(12.17, 14.02)
Vomiting	781	3.35	(3.12, 3.61)	3.19	(2.97, 3.44)
Abdominal distension	662	11.82	(10.92, 12.79)	11.21	(10.35, 12.13)

## Discussion

This work studies the adverse drug event relationship between pancreatitis and the GLP-2 analog teduglutide. A disproportionality analysis was conducted using data obtained from the FAERS database from over four years of spontaneous drug reaction reports, including 11,696 cases of teduglutide adverse events and 79 cases of pancreatitis reports associated with teduglutide therapy. Based on the computation of statistics such as ROR, PRR, EBGM, and IC2, our analysis suggests that the risk for developing pancreatitis is around 3.7 times higher in patients using teduglutide and at least almost three times higher at a 95% confidence level.

Our analysis also studies other common adverse effects of teduglutide therapy, such as vascular device infection, weight changes, abdominal pain, nausea, and diarrhea. We also see a statistically significant increase in these adverse drug events associated with teduglutide as a result of our analysis, which confirms reports of the adverse drug events in prior clinical studies of teduglutide [6-8].

The finding of a significant association between pancreatitis and teduglutide may be surprising [12]. This is because GLP-2 receptors are not known to be expressed in the pancreas [12]. Nonetheless, various clinical reports have raised concerns about patients on teduglutide developing pancreatitis [6,11,14,15], and our findings reveal that pancreatitis is in fact statistically linked to teduglutide use.

Several clinical studies have documented elevated pancreatic enzymes as an adverse effect of teduglutide therapy [10-13]. Elevated pancreatic enzymes such as amylase and lipase are considered a specific indicator of acute pancreatitis [13]. However, in the setting of teduglutide therapy, multiple cases have been observed which show elevated pancreatic enzymes without other clinical signs of acute pancreatitis [12,13]. Therefore, it is unclear whether the adverse drug event of elevated pancreatic enzymes is related to the adverse drug event of pancreatitis for teduglutide therapy. In our study, we were unable to study the relationship between elevated pancreatic enzymes with teduglutide since these adverse events were not reported in the FAERS database.

It is noteworthy that a significant adverse drug event association with pancreatitis has been previously established by various works for the related drug class of GLP-1 receptor agonists in the setting of pharmacologic treatment of type 2 diabetes mellitus (T2DM). To this end, multiple works have conducted disproportionality analyses of this association using the FAERS database and other retrospective analyses [19-22] which confirm clinical reportings of an increased risk of pancreatitis with GLP-1 agonist use. Given that GLP-1 and GLP-2 are structurally related by a common precursor protein [23], proglucagon, it is possible that the increased pancreatitis risk associated with GLP-1 and GLP-2 agonists may be mediated by similar biological mechanisms.

This study has several limitations that could be improved upon in further work. One drawback is that confounding variables may exist in the correlation between teduglutide and pancreatitis that are not accounted for in this study. For example, our study methodology identifies vascular device infection as an adverse drug event of teduglutide, although this adverse drug event is likely more appropriately attributed to the use of parenteral support in these patients [4]. Therefore, this study could be improved by controlling for various possible confounding variables, including the use of parenteral support, other treatments or medications, and surgical interventions related to SBS. A related point is that this study is not able to offer a plausible biological mechanism by which teduglutide causes pancreatitis. Indeed, this lack of a plausible biological mechanism may explain why this adverse drug event relationship has been relatively understudied in the literature [12]. Finally, the data collected in the FAERS database are based on spontaneous reports of adverse drug events and may not be able to offer a truly representative sample of the risks associated with adverse drug events due to selection bias.

## Conclusions

While several clinical studies have previously found elevated pancreatic enzymes and pancreatitis to be an adverse outcome of teduglutide in isolated cases and small trials, our study gives the first systematic disproportionality analysis using the FAERS database. Our study finds that there is a statistically significant adverse drug event association between teduglutide therapy and pancreatitis. We therefore highlight the importance of monitoring for possible pancreatitis via lab tests for pancreatitis and imaging studies such as CT, MRI, or ultrasound when treating SBS patients with teduglutide.
